# Enhanced Lycopene Production by UV-C Irradiation in Radiation-Resistant *Deinococcus radiodurans* R1

**DOI:** 10.4014/jmb.2009.09013

**Published:** 2020-10-08

**Authors:** Chang Keun Kang, Jung Eun Yang, Hae Woong Park,, Yong Jun Choi

**Affiliations:** 1 School of Environmental Engineering, University of Seoul, Seoul 02504, Republic of Korea; 2 Department of Advanced Process Technology and Fermentation, World Institute of Kimchi, Gwangju 61755, Republic of Korea

**Keywords:** *Deinococcus radiodurans*, metabolic engineering, lycopene, UV-C radiation

## Abstract

Although classical metabolic engineering strategies have succeeded in developing microbial strains capable of producing desired bioproducts, metabolic imbalance resulting from extensive genetic manipulation often leads to decreased productivity. Thus, abiotic strategies for improving microbial production performance can be an alternative to overcome drawbacks arising from intensive metabolic engineering. Herein, we report a promising abiotic method for enhancing lycopene production by UV-C irradiation using a radiation-resistant Δ*crtLm*/*crtB*^+^*dxs*^+^ *Deinococcus radiodurans* R1 strain. First, the onset of UV irradiation was determined through analysis of the expression of 11 genes mainly involved in the carotenoid biosynthetic pathway in the Δ*crtLm*/*crtB*^+^*dxs*^+^ *D. radiodurans* R1 strain. Second, the effects of different UV wavelengths (UV-A, UV-B, and UV-C) on lycopene production were investigated. UV-C irradiation induced the highest production, resulting in a 69.9% increase in lycopene content [64.2 ± 3.2 mg/g dry cell weight (DCW)]. Extended UV-C irradiation further enhanced lycopene content up to 73.9 ± 2.3 mg/g DCW, a 95.5% increase compared to production without UV-C irradiation (37.8 ± 0.7 mg/g DCW).

## Introduction

Lycopene is a carotenoid, naturally occurring in red-colored fruits and vegetables, and has long been of great interest due to its antioxidant and anticancer properties [1-3]. As the industrial demand for lycopene increases, great effort has been made to develop improved microbial strains capable of lycopene production through classical metabolic engineering. Recently, the development of genetically modified *Saccharomyces cerevisiae* and *Escherichia coli* strains capable of producing 2.37 g/l [73.3 mg/g dry cell weight (DCW)] and 3.52 g/l (50.6 mg/g DCW) of lycopene, respectively, was achieved through the engineering of lipid biosynthetic pathways followed by optimizing cofactor and precursor availability [4, 5]. Most recently, the highest reported lycopene content of up to 203.5 mg/g DCW was successfully achieved in the metabolically engineered *Deinococcus radiodurans* R1 strain using waste resources [6].

To date, conventional metabolic engineering, including genome engineering, multi-omics, and metabolic flux analysis, has been a very efficient approach for the development of industrial microbial strains and has successfully contributed to the fermentative production of desired bioproducts. However, the current strategies for microbial strain development still require considerable time and effort for the prevention of adverse effects caused by intensive genetic manipulation such as genetic instability, imbalanced carbon flux, and metabolic burdens [7-9]. Although several strategies can be used to reduce adverse effects through *in silico* simulation and multi-omics analysis, their practical application is very limited due to the complexity of the metabolic network. Furthermore, due to the irreversibility and labor aspect of extensive genetic engineering, researchers have attempted to enhance the production of carotenoids by controlling abiotic factors instead. As an example, applying an appropriate level of stress can have a positive effect on production performance. The total carotenoid content of *Haematococcus pluvialis* increased up to 32.0 mg/g DCW by exposure to various stressors such as reduced nitrogen and phosphorus supplementation, salinity, and strong light. Moreover, the expression of genes involved in the carotenoid biosynthetic pathway increased under stress conditions [10]. In another study, the temperature was also used as a carotenoid production-enhancing abiotic factor. More than a 2.1-fold increase in β-carotene (264 ± 1 mg/l) was successfully achieved in *Rhodotorula glutinis* by temperature control without metabolic engineering approaches [11].

In recent years, ultraviolet (UV) radiation has been extensively studied as a stressor increasing metabolic flux towards the carotenoid pool. This originated from the hormesis-induced stress response mechanisms, whereby UV radiation triggers the production of reactive oxygen species (ROS), which would lead to an increase in carotenoid production. A marked increase in carotenoids, including β-carotene, astaxanthin, and lutein, was observed in microalgae such as *Dunaliella* spp., *Tetraselmis suecica,* *Nitzschia closterium*, *Isochrysis zhangjiangensis*, and *H. pluvialis* exposed to UV radiation [12-15]. This is consistent with previous studies on the effects of UV radiation on lycopene content in tomatoes [16, 17].

The red-pigmented extremophilic bacterium *D. radiodurans* R1 has great potential for use as a platform strain for the production of carotenoids, including phytoene and lycopene [6, 18, 19]. This bacterium not only harbors the carotenoid biosynthetic pathway [20], but also has a high concentration of NAD (P)H [21], an important metabolite for the production of lycopene. Moreover, unlike other microorganisms that are highly vulnerable to UV radiation, the *D. radiodurans* R1 strain is well known for its notable resistance to radiation, including UV-C (> 500 J/m^2^) [22].

**Fig. 1:**
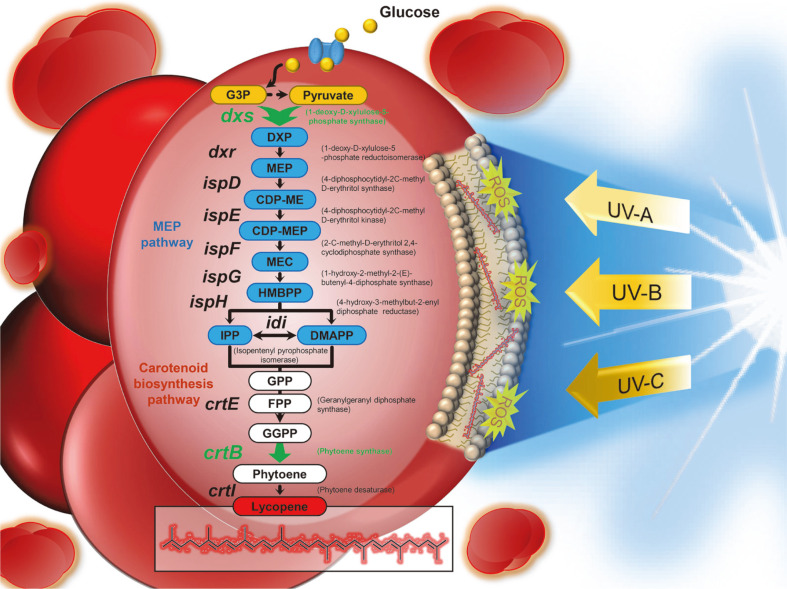
Lycopene biosynthesis-related genes in the MEP pathway and carotenoid biosynthesis pathway of *D. radiodurans* R1. The green arrows indicate plasmid-borne overexpression. Abbreviations: G3P, glyceraldehyde-3- phosphate; DXP, 1-deoxy-D-xylulose-5-phosphate; MEP, 2-C-methyl-D-erythritol-4-phosphate; CDP-ME, 4-diphosphocytidyl- 2-C-methyl D-erythritol; CDP-MEP, 4-diphosphocytidyl-2-C-methyl D-erythritol 2-phosphate; MEC, 2-C-methyl-Derythritol 2,4-diphosphate; HMBPP, (E)-4-hydroxy-3-methylbut-2-enyl pyrophosphate; IPP, isopentenyl diphosphate; DMAPP, dimethylallyl diphosphate; GPP, geranyl diphosphate; FPP, farnesyl diphosphate; GGPP, geranylgeranyl diphosphate; UV, ultraviolet; ROS, reactive oxygen species.

These observations led us to investigate the effects of UV radiation as a stressor for the enhanced production of lycopene using a radiation-resistant extremophilic microorganism, *D. radiodurans* R1. Previously, we reported the genetically engineered *D. radiodurans* R1 strain capable of producing lycopene with high yield by metabolic engineering of the carotenoid biosynthetic pathway [6]. In this strain, the crtLm gene encoding lycopene cyclase was first removed to block the conversion of lycopene to γ-carotene. Then, phytoene synthase and 1-deoxy-D-xylulose 5-phosphate synthase encoded by the crtB gene and dxs gene, respectively, were overexpressed to reroute the metabolic flux towards lycopene. In this study, the effect of UV irradiation on microbial lycopene production was extensively explored through comparative analysis of the mRNA expression levels of genes involved in the carotenoid biosynthetic pathway in previously reported Δ*crtLm*/*crtB*^+^*dxs*^+^ *D. radiodurans* R1 strain (Fig. 1). The onset and exposure times of UV-C irradiation for increasing lycopene content were then established.

## Materials and Methods

### Strain and Culture Medium

The engineered *D. radiodurans* strain (Δ*crtLm*/*crtB*^+^*dxs*^+^) [6] was grown in TGY or semi-defined media. TGY medium contained 0.5% tryptone, 0.1% glucose, and 0.3% yeast extract. The semi-defined medium contained 10 g/l glucose, 30 g/l Na_2_HPO_4_, 15 g/l KH_2_PO_4_, 9.9 g/l (NH_4_)_2_SO_4_, 5 μM MnCl_2_, 0.8 mM MgCl_2_, 0.18 mM CaCl_2_, 1%BME vitamins 100× solution (Sigma Aldrich, USA), 50 mg/l L-cysteine, 25 mg/l L-histidine, 25 mg/l L-methionine, and 1 g/l yeast extract.

### Shake-Flask Cultivation and UV Irradiation

For the production of lycopene, fresh colonies were inoculated into 14 ml culture tubes containing 4 ml TGY broth and were grown for 24 h. The seed culture was used to inoculate a 250 ml flask containing 80 ml of semi-defined medium (initial OD_600_ = 0.05), followed by 72 h of shake-flask culture. All cultures were incubated at 30 °C with shaking at 0.44 ×*g* under complete dark condition. Chloramphenicol (3 μg/ml) was added to the medium to maintain plasmid stability.

For UV-induced overproduction of lycopene, a shaking incubator (JS Research, Korea) was equipped with the Philips MASTER Actinic BLhilips TL-D 15W WEATHERING, and Philips TUV 15W lamp (Philips, The Netherlands) for irradiation with UV-A (365 nm), UV-B (290 nm), and UV-C (253 nm), respectively. The shaking incubator was 500 mm wide, 500 mm deep, and 495 mm high. Bacterial cells in the early- or mid-exponential growth phase were transferred to 250 ml quartz flasks and irradiated by UV in a shaking incubator at 30°C with shaking at 200 rpm. The distance between the lamp and flasks was approximately 350-400 mm.

### Analytical Methods

Cell growth was monitored by measuring the absorbance at 600 nm (OD_600_) using an Epoch microplate spectrophotometer (Biotek, USA).

For the analysis of lycopene content, cells were harvested from 1 ml of medium by centrifugation at 16,000 ×*g* for 1 min and then washed twice with distilled water. Dry cell weight was measured after vacuum lyophilization, and lycopene was extracted from the lyophilized cells. Cells were sonicated for 1 min in 0.5 ml of methanol, and then 0.5 ml of acetone was added to the sonicated cells. After incubation at 55 °C for 15 min, the suspension was centrifuged at 16,000 ×*g* for 1 min, and the supernatant containing lycopene was filtered using a PTFE 0.2 μm pore size syringe filter.

The extracts were analyzed using a high-performance liquid chromatography (HPLC) system (Agilent 1260 InfinityII, Agilent Corporation, USA) equipped with a Zorbax Eclipse XDB-C18 column (4.6 × 250 mm, Agilent) as described previously [6].

Glucose concentration in the medium was measured as described previously [6] using an HPLC system equipped with a refractive index detector (Agilent 1260 InfinityII). The MetaCarb 87H column was eluted with 0.005 NH_2_SO_4_ at 60°C at a flow rate of 0.5 ml/min.

### Quantitative Real-Time PCR

Total RNA was isolated from the harvested cells using the RiboEx reagent (GeneAll, Korea) according to the manufacturer’s instructions. DNA was removed by treatment with RNase-free DNase I (Takara, Japan). Total RNA concentration was quantified by measuring the A_260_/A_280_ ratio using an Epoch microplate spectrophotometer with a Take3 Plate (Biotek).

A 1 μg sample of total RNA was used to synthesize cDNA with random primers using a SuperiorScript III cDNA Synthesis Kit (Enzynomics, Korea). The relative expression levels of 11 genes (*dxs, dxr, ispD, ispE, ispF, ispG, ispH, idi, crtE, crtB*, and *crtI*) were analyzed by quantitative real-time (qRT) PCR using the Bio-Rad CFX Connect Real-Time System (Bio-Rad, USA) with RbTaq Fast qPCR 2X PreMIX (Enzynomics, Korea). All primers used in the qRT-PCR are shown in Table 1. For each reaction, 1 μl of each primer (10 μM) and 10 μl of RbTaq Fast qPCR 2X PreMIX were added in a total volume of 20 μl. The qRT-PCR protocol included 95°C for 30 sec, followed by 35 cycles at 95°C for 10 sec and 60°C for 10 sec. The *dr1343* encoding glyceraldehyde 3-phosphate dehydrogenase was used as an internal control for normalization.

## Results and Discussion

### Determination UV Irradiation Onset by Comparative Analysis of mRNA Expression Levels

**Fig. 2:**
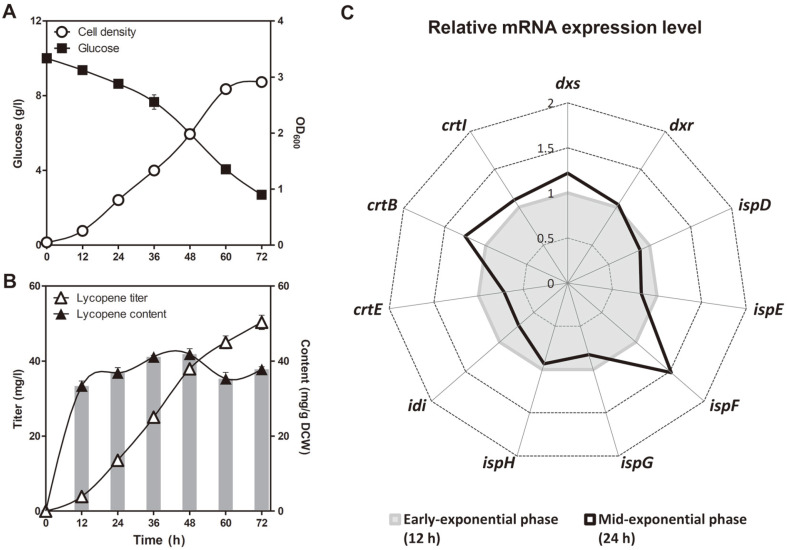
Shake-flask culture profiles and relative mRNA expression levels of lycopene biosynthesis-related genes of Δ*crtLm*/*crtB*^+^*dxs*^+^ *D. radiodurans* R1 grown in the dark. Time-course profiles of (**A**) cell density and glucose consumption, (**B**) lycopene titer and content. Error bars represent the standard deviations of experiments conducted in triplicate. (**C**) Radar plot describing the relative mRNA expression level of lycopene biosynthesis-related genes at the midexponential phase (24 h) versus at the early exponential phase (12 h). The mean of three biological replicates is shown.

A previously constructed lycopene-overproducing Δ*crtLm*/*crtB*^+^*dxs*^+^ *D. radiodurans* R1 strain [6] was employed as the base strain to explore the effect of UV irradiation on microbial lycopene production. As can be seen in Figs. 2A and 2B, during the early exponential growth phase (12 h), lycopene content tended to rapidly increase up to 33.4 ± 1.3 mg/g DCW, but no significant increase was observed in the mid-exponential growth phase (24 h), which is consistent with our previous study [6]. In addition, the expression of the *idi* (isopentenyl diphosphate isomerase gene) and *crtE* (geranylgeranyl diphosphate synthase gene) genes, which encode the major rate-limiting enzymes in the carotenoid biosynthetic pathway [5, 7, 23], was lower at 24 h than at 12 h (Fig. 2C). This is because the carbon flux of carotenoid biosynthesis shifted towards biomass formation as it entered the mid-exponential growth phase. Therefore, the mid-exponential growth phase (24 h) was selected for the onset of UV irradiation.

### Effects of UV-C Irradiation on Lycopene Production

**Fig. 3:**
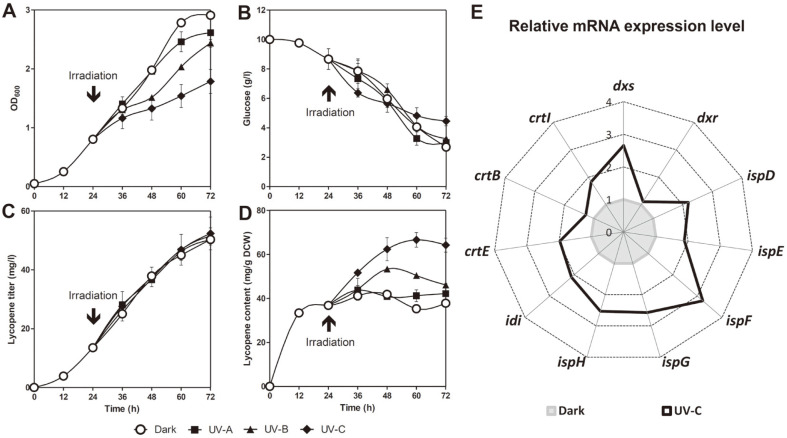
Effect of UV irradiation at mid-exponential growth phase on lycopene production and gene expression. Time-course profiles of (**A**) cell density, (**B**) glucose consumption, (**C**) lycopene titer, and (**D**) content. Error bars represent the standard deviations of experiments conducted in triplicate. (**E**) Relative mRNA expression level of lycopene biosynthesisrelated genes in UV-C-irradiated cells versus that of dark-grown cells at 36 h. The mean of three biological replicates is shown. Cells were dark-grown or irradiated by UV-A, B or C at the mid-exponential phase (24 h) for 6 h using 15W lamp in the shaking incubator.

The positive effect of UV irradiation on carotenoid biosynthesis has been previously reported in various algal species [12-15]. Based on these studies, in order to determine the type of UV rays, Δ*crtLm*/*crtB*^+^*dxs*^+^ *D. radiodurans* R1 at the mid-exponential growth phase (24 h) was irradiated with three different wavelengths (UV-A (365 nm), UV-B (290 nm), and UV-C (253 nm)) for 6 h. As can be seen in Fig. 3A and 3B, the cell growth rate decreased proportionally with shorter UV wavelengths, despite no significant changes in the glucose uptake rate. Although the lycopene titer steadily increased to 50.3 ± 0.9 (UV-A), 51.6 ± 2.8 (UV-B), and 52.4 ± 5.6 mg/l (UV-C), it did not differ significantly from the dark conditions (50.3 ± 1.9 mg/l) (Fig. 3C). However, the lycopene content significantly increased by 11.6% (42.2 ± 4.7 mg/g DCW) and 22.0% (46.1 ± 1.4 mg/g DCW) under UV-A and UV-B irradiation, respectively, compared to the dark condition. Furthermore, the highest value of 64.2 ± 3.2 mg/g DCW was achieved through UV-C irradiation, a 69.9% increase in lycopene content compared to the same strain under the dark condition at the endpoint of cultivation (37.8 ± 0.7 mg / g DCW) (Fig. 3D). Thus, it was concluded that UV-C irradiation was optimal for increasing lycopene content.

Next, in order to investigate the mechanism through which UV-C irradiation increases lycopene production, the mRNA expression levels of the 11 carotenoid biosynthesis-related genes were comparatively analyzed in the Δ*crtLm*/*crtB*^+^*dxs*^+^ *D. radiodurans* R1 strain after UV-C irradiation. As shown in Fig. 3E, the mRNA expression levels of analyzed genes were upregulated by UV-C irradiation. In particular, a more than 2-fold increase was observed in the expression of *crtE, dxs*, and *idi*, which are responsible for major carotenoid biosynthetic pathway rate-limiting steps, as well as in that of *ispDEFGH* genes involved in the 2-C-methyl-D-erythritol-4-phosphate (MEP) pathway. The upregulation of genes responsible for carotenoid biosynthesis can be interpreted as a defense mechanism for reducing ROS generated by UV-C irradiation [24-26]. That is, their increased expression leads to increased carbon flux through the corresponding pathway, thus enhancing the biosynthesis of ROS-scavenging antioxidants. The current results indicate that the redirection of the carbon flux to the carotenoid biosynthetic pathway was successfully achieved through UV-C irradiation at the intermediate exponential growth stage, resulting in increased lycopene synthesis.

### Enhanced Lycopene Production by Long-Term UV-C Irradiation

**Fig. 4:**
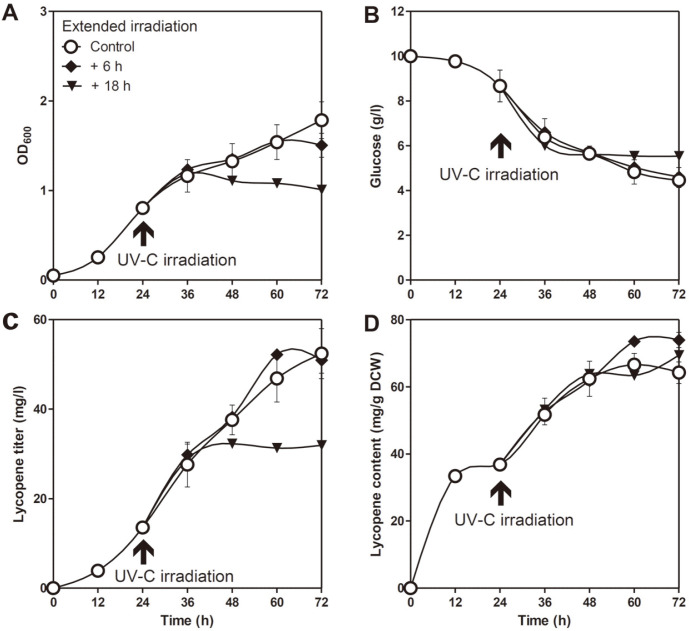
Optimization of UV-C irradiation duration. Time-course profiles of (**A**) cell density, (**B**) glucose consumption, (**C**) lycopene titer, and (**D**) content. Cells were irradiated by UV-C for 6 h (control), 12 h (control + 6 h), or 24 h (control + 18 h). UV-C irradiation was initiated at the mid-exponential phase (24 h) using a 15 W lamp in the shaking incubator. Error bars represent the standard deviations of experiments conducted in triplicate.

Since UV-C irradiation enhanced lycopene production in the *D. radiodurans* R1 strain as described above, we hypothesized that extended irradiation might further upregulate lycopene production. To examine whether lycopene production was further enhanced by long-term exposure to UV-C rays, Δ*crtLm*/*crtB*^+^*dxs*^+^ *D. radiodurans* R1 was irradiated for an additional 6 h or 18 h. As shown in Figs. 4A and 4B, the growth rate and glucose uptake rate were significantly reduced following 18 h of additional irradiation. This is probably due to severe cellular damage by excessive UV irradiation. Although the lycopene titer obtained following an additional 6 h of irradiation (51.0 ± 2.9 mg/l) was similar to that obtained without additional UV-C irradiation (52.4 ± 5.6 mg/l), lycopene content increased by 15.1% (73.9 ± 2.3 mg/g DCW) after 6 h (Figs. 4C and 4D). These results demonstrated that an appropriate level of additional UV-C treatment could further increase lycopene content.

**Fig. 5:**
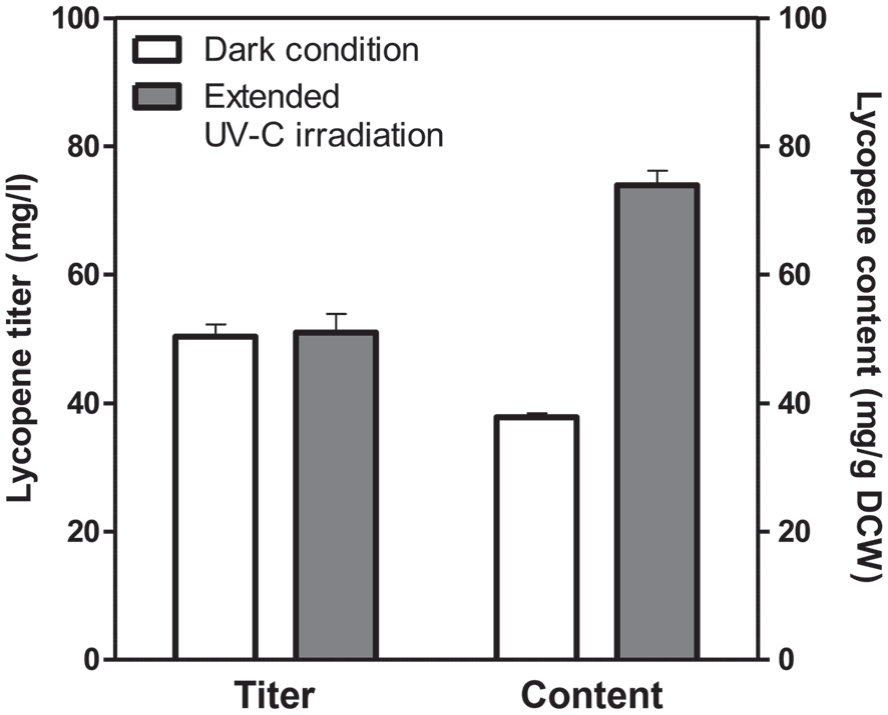
Lycopene titer and content of the Δ*crtLm*/*crtB*^+^*dxs*^+^ *D. radiodurans* R1 strain after 72 h of shake-flask culture. Cells were grown in the dark condition or under extended UV-C irradiation. UV-C irradiation was initiated at the mid-exponential phase (24 h) using a 15W lamp and lasted for 12 h in the shaking incubator. Error bars represent the standard deviations of experiments conducted in triplicate.

In conclusion, we reported, for the first time, a promising strategy for improving the microbial production of lycopene through UV-C irradiation using the lycopene-producing extremophilic bacterium Δ*crtLm*/*crtB*^+^*dxs*^+^ *D. radiodurans* R1 strain. After irradiation with UV-C rays at the mid-exponential growth phase, the lycopene titer and content increased by 4.2% (52.4 ± 5.6 mg/l) and 69.9% (64.2 ± 3.2 mg/g DCW), respectively, compared to those without UV-C irradiation. Finally, lycopene content was further improved by 15.1% (73.9 ± 2.3 mg/g DCW) following extended UV-C irradiation for 6 h, resulting in a 95.5% increase compared to cells grown without UV-C (37.8 ± 0.7 mg/g DCW) (Fig. 5).

The method for improving lycopene production through UV-C irradiation developed in this study has advantages in terms of cost-effectiveness and reproducibility. In addition, the *D. radiodurans* R1 strain used in this study has strong resistance not only to UV rays but also to various external stresses such as temperature, salinity, and desiccation. Thus, it can be widely applied in the production of various chemicals and compounds using different abiotic factors without the unwanted properties caused by intensive genetic engineering during strain development. Further studies are currently underway to improve UV-C resistance through the adaptive evolution approach, which is also expected to enhance lycopene productivity and overcome growth retardation caused by UV-C irradiation.

## Figures and Tables

**Table 1:** Primers used in this study.

Primer	Sequence (5´→3´)
dr1343-F	caacgacctgaccgacaacc
dr1343-R	ggctgctttcgtcgtactcc
dxs(dr1475)-F	ctgcgcgggatgctcaagta
dxs(dr1475)-R	atttcaggtccggccacgtc
dxr(dr1508)-F	gaagcatccgtcgtggagca
dxr(dr1508)-R	cgtagaggctggcacactcc
ispD(dr2604)-F	cctctgggcggtgcaaacac
ispD(dr2604)-R	gtcgtcggtggcagcgtact
ispE(dr2605)-F	cctcggcctctcggtcctt
ispE(dr2605)-R	aggccgaatttccagctcgt
ispF(dr0230)-F	gtgaacgtcgctctcgtggt
ispF(dr0230)-R	cagcaggcagaccgagcag
ispG(dr0386)-F	gagcaagcacgccaacatcg
ispG(dr0386)-R	ttcagcgtcgtcagcagctt
ispH(dr2164)-F	gcgtcgtcatggcgattcag
ispH(dr2164)-R	cgctccaccaccgtgtgatt
idi(dr1087)-F	tcttgcagcgggttgaggtg
idi(dr1087)-R	cgcggcgcagtttatgctc
crtE(dr1395)-F	ttcgggacgacgtgctcaac
crtE(dr1395)-R	gatcagggtgcgcttgcctt
crtB(dr0862)-F	caggccgtattcgtcgagca
crtB(dr0862)-R	aactcggtcaggcgatgcag
crtI(dr0861)-F	agcaggctcatgctttcgct
crtI(dr0861)-R	cgactgggcgaacacctacc
